# Experiences in Developing and Implementing Health Clubs to Reduce Hypertension Risk among Adults in a South African Population in Transition

**DOI:** 10.1155/2012/913960

**Published:** 2012-08-16

**Authors:** Thandi R. Puoane, Lungiswa Tsolekile, Ehimario U. Igumbor, Jean M. Fourie

**Affiliations:** ^1^School of Public Health, University of the Western Cape, Private Bag X17, Bellville 7535, South Africa; ^2^Chronic Diseases of Lifestyle Research Unit, Medical Research Council, P.O. Box 19070, Tygerberg 7505, South Africa

## Abstract

Chronic noncommunicable diseases (NCDs) are increasing substantially as a cause of death and disability in all strata of the South African society, particularly among the urbanised poor. Hypertension is a risk factor for many of these diseases and becoming a burden in a growing population in a Cape Town township, Khayelitsha. To alleviate healthcare demands at clinics in this area, a health club was initiated and community health workers (CHWs) were trained to empower community members about NCDs and create public awareness. After training, a health club was initiated. Three months after initiation of the health club, 76 participants had been recruited of whom 22 were regular attenders. New members joined the health club weekly. Anthropometric and blood pressure measurements were taken, and various hypertension topics were covered at the club meetings which included healthy behaviours, such as the benefits of being physically active and eating healthy. Nutrition education sessions based on the South African food-based dietary guidelines were also held. Consequent to the initial group that was established, two more clubs were formed in the area. Health clubs are sustainable and culturally appropriate when facilitated by local people who have an insight and deeper understanding of the culture and environment of the people they serve.

## 1. Background and Rationale 

Evidence that patterns of human health and disease are predicated on socioeconomic and environmental conditions is compelling [1]. As a population that has witnessed significant transitions over the past few decades, black South Africans exemplify how overall health status is moulded by political and socioeconomic changes. Not until the first democratic elections in 1994 did the enshrined structural inequities limiting their freedom of natural growth and potential to participate more equally in the economic and social life of South Africa, give way to a freedom to choose where to live and work. With the end of the oppressive history of colonisation and apartheid, most black South Africans have had to grapple with a new set of challenges affecting their health. Contributing to a recent Lancet review on “Health in South Africa,” Coovadia et al. [2] noted that “the distinctive features of South Africa's history that account for the current health problems include racial and gender discrimination, income inequalities, migrant labour, the destruction of family life, and persistent violence spanning many centuries but consolidated by apartheid in the 20th century.” Indeed, even a cursory analysis of the country's current health profile will not miss the simultaneous occurrence of epidemic infectious diseases, perinatal and maternal disorders, injuries, and chronic noncommunicable diseases (NCDs) [3]. 

Chronic NCDs including hypertension is growing substantially as a cause of death and disability in all strata of the South African society, but most predominantly among poor people living in urban settings [4, 5]. This pattern mirrors the prevalence of risk factors for hypertension and underlying determinants including demographic and nutritional transitions arising from socioeconomic development and increasing globalisation. Steyn et al. [6] analysed results of the South African Demographic and Health Survey (SADHS) to identify population groups with a high prevalence and poor control of hypertension in the country. They showed that “hypertension risk was lowest in rural blacks and significantly higher in obese black women than in women with a normal body mass index” [6]. High risk of hypertension was associated with education below tertiary level, older age groups, being overweight and obese, excess alcohol use, and a family history of stroke and hypertension. 

The growing trend has been exacerbated by the rapid increase in urbanization, notably the unprecedented levels of migration from century-old traditional lifestyles in rural areas to the large periurban settlements of cities. This migration contributes to the increasing inequities between the rich and poor in respect of hypertension risk; people who live in poverty tend not to benefit from the higher living standards of urban life. Urbanization has an effect on almost all aspects of the migrant's lifestyle contributing to increasing risk of hypertension and other NCDs. These include the influence on the migrant's diets, level of physical activity, and use of alcohol and tobacco products. 

In many developing countries including South Africa there has been a shift in dietary intake from a traditional diet high in fibre to a more western diet that is high in fat and refined carbohydrates [7]. In addition, data have shown that South Africans consume more salt than the recommended maximum of 6 g/day [8].

Regarding physical activity, urban dwellers more often make use of motorized transportation, accessible public transport, and thus less walking or less labour-intensive work, while having more sedentary leisure activities like watching television than rural dwellers. Studies have shown that even moderate intensity activity is protective [9–11]. According to Norman and colleagues [12], an estimated 3.3% of total deaths in South Africa was due to physical inactivity. 

The contributing factors cited as driving the nutrition transition include the limited availability of affordable healthy food in poorer periurban areas, combined with the increased availability of fast foods that are high in fats and sugar [13]. Other contributing factors to physical inactivity are limited outdoor space, high rates of street violence, and cultural beliefs and practices [13]. 

Along with changes in dietary intake and physical inactivity, obesity is also prevalent in South Africa. Urban African women had the highest rate compared to other racial groups [14, 15]. Some studies have reported that obesity among African women was associated with being affluent, attractive, and healthy [16, 17], and although they were aware of the consequences of being overweight, they perceived weight loss as a source of stigma and a sign of disease, particularly of HIV/AIDS [13, 18]. 

Despite awareness of these different factors, the focus of hypertension prevention remains largely on advocating change in individual lifestyles [13]. Satcher [19] has argued that “hypertension control is an appropriate goal and certainly the elimination of disparities in hypertension control must take place in the context of physical and social environments and human behaviour.” Approaches that recognise the centrality of individual behaviour and community-based health promotion, and which exploit local initiative and opportunities, are required. 

## 2. The Development of a Health Club in Khayelitsha

Khayelitsha (Meaning “New Home”) is a township for black Africans located on the Cape Flats, approximately 35 km outside Cape Town. This is the fastest growing township in South Africa, which mainly consists of informal settlements. However, a third of the houses are formal brick structures pointing to the relative affluence and better socioeconomic status of some parts of Khayelitsha compared to others. It further suggests the increasing formalization of settlements within Khayelitsha as more permanent residential structures now exist [20]. The population of Khayelitsha is continuously growing with newcomers arriving mainly from the adjacent Eastern Cape province. Although health clinics serve the community, the staff members are not always able to meet the preventive care or needs of their chronic patients. Therefore, the help of community health workers (CHWs) were introduced to assist with the current shortfalls in human resources [21].

A nongovernmental organization invited us to collaborate with them by utilizing local CHWs to increase the community's awareness about the risk factors for NCDs. Initially, we worked with CHWs, who were also at risk for NCDs. A large percentage (97%) of them had a body mass index (BMI) > 30 kg/m^2^ [16]. Jointly, a training programme was developed to equip CHWs with knowledge and skills to assist them to work with the community. Community health workers were expected to develop and implement the interventions for primary prevention of NCDs in the community for increasing awareness about risk factors and prevention strategies, upon completion of the training program. Numerous activities for increasing awareness about risk factors for NCD were implemented by the CHWs and these included organising fun walks in the township, and staging a drama to disseminate messages about causes and prevention of hypertension and diabetes.

Community members in the township recognised and appreciated the activities of the CHWs and requested regular weekly meetings where they could meet and be educated about risk factors and preventive measures for hypertension and diabetes, which was seen to be affecting many people in this community. This led to the development of the health club in the Khayelitsha township.

The health club was developed by the CHWs who had received training on the primary prevention of cardiovascular disease during October 2001 to October 2002 [21]. Training of CHWs on facilitation of health clubs with a focus on exercise, diet, and anthropometry (blood pressure, weight, and height) was conducted over six months and commenced in 2005. On completion of the training, each CHW recruited five people around their place of residence to join the health club. CHWs also chose the name of the health club “*Masiphakame ngempilo yethu*” meaning “let us stand up for our health.” The process of the development of a health club has been described by Puoane et al. [21].

**Figure 1:**
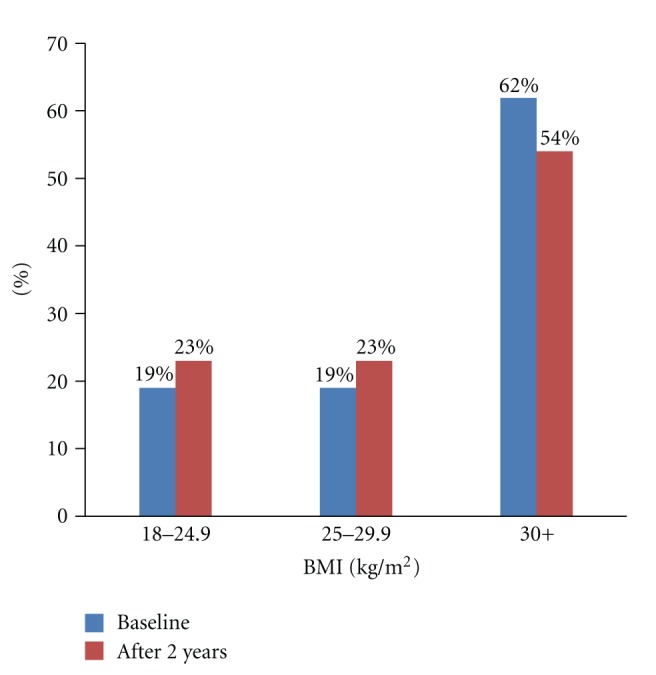
Changes in body mass index among health club participants over a 2 year period.

On the first day of the club meeting, only eight participants registered and all were females, subsequently the numbers increased weekly. By the end of three months, 76 participants had been recruited and every week new members joined the health club. Participants were males and females between ages 30 and 65 years. Eighty-two percent of the participants had a BMI > 25, falling in the overweight to grossly obese categories (Figure 1). Two years after the intervention, there was a reduction in the number of participants who were obese (i.e., BMI > 30 kg/m^2^). However, Overweight (25–29.9 kg/m^2^) and obesity (30 kg/m^2^) still remain a problem in this population. Although there were 76 participants who initially joined the health club, only 22 attended the sessions regularly (Table 1).

Anthropometric measurements including weight, height, and blood pressure were collected at baseline (Table 2). The sessions were held once a week and monthly anthropometric measurements were collected. Nutrition education sessions were held once a month and cooking demonstrations were incorporated into the sessions. T-shirts with the name of the club were issued to all new members after recruitment.

During the past 6 years, four fun walks, two diabetes workshops, and three community-based introductory sessions were held to create awareness. Two additional clubs have been formed since inception and additional training was offered to the CHWs. There have been 34 referrals to the nearest clinic for medical attention.

## 3. Facilitation of the Health Club

During the health club meeting sessions, CHWs facilitated the physical activity sessions, followed by the cooking demonstration, tasting of food, and sharing of recipes to try them out at home. At each cooking demonstration, participants evaluated the meals for palatability and recipes were issued to all participants. Health talks were given at every session and guided by the food-based dietary guidelines (FBDGs, Box 1) which were developed by the South African Department of Health [22]. This was done to ensure that the messages conveyed at the health clubs were in agreement with those of the Department of Health. Once a month all club members joined a 2–5 km walk around the township. 

Various topics were covered and these included the following: (1) basics on the aetiology of hypertension and diabetes, (2) signs and symptoms of these conditions, (3) prevention of the conditions, (4) importance of medication, and (5) the role of nutrition and physical activity in the prevention of hypertension and diabetes. In addition, the FBDGs were used to influence adoption of healthy living among participants. 

## 4. Challenges in Implementing a Health Club 

The implementation of a health club in a resource-poor setting had numerous challenges. Data from health club records show that 76 participants were initially recruited, however, only 22 were regular attendees and two years later the number had increased to 30. This slow growth in numbers can be explained by the constant movement of people living in these settlements. Informal settlements serve as temporal accommodation while people are waiting to be allocated to formal housing. This constant movement of participants results in regularly having new participants. Thus, monitoring progress especially at an individual level becomes difficult. Implementation of such initiatives in mobile communities can be a challenge when conducting an evaluation. 

Competing personal priorities also limited people from attending the health club regularly. Census data show that 51% of the population in Khayelitsha is unemployed, with more males being unemployed as compared to females [23]. Furthermore, a majority of the health club members moved to the city in search of employment. Therefore, when employment opportunities arose, they often left the health club. 

The program ran during the day, thus limiting participation of the economically active individuals. Furthermore, employed residents who may have had an interest in the health club could not participate. This further explains why the club members tended to be older.

Mbombo [24] reported that although hypertension is a common condition among poor South Africans, a large percentage is undiagnosed due to a lack of routine physical checkups; people often seek medical aid only when they feel pain. Poor health-seeking behaviour in this population explains the inability to attract healthy individuals to partake in interventions that focus on prevention of diseases.

This paper demonstrates that education on lifestyle modification focusing on healthy eating and physical activity is possible even in resource-limited settings. However, lack of a supportive environment hampered the maintenance of healthy practices. For example, in a study to identify environmental risk factors for NCDs conducted in Khayelitsha, it was reported that generally there was a shortage of healthy, low-fat food and little fresh fruit and vegetables available [25]. In addition, local shops sold cheap fatty foods, while street vendors often sold fatty meat and sausages [25]. Thereby confirming that to promote healthy living in a disabling environment complicates decisions on healthy choices. 

Although the primary focus of the health club was physical activity and healthy eating, it yielded unexpected outcomes. The health club also provided a supportive environment where participants could obtain emotional support. As reported in the testimonies (Box 2), participants saw the health club as a place of sharing problems with people who might be experiencing the same problems. Studies show that individuals who face more stressful events are more likely to benefit from the buffering effects of social support [26]. Other studies suggest that social support may add more “value” to those who face increased stressors [27].

## 5. Conclusions

The experience in implementing a health club in South Africa lends learning for other developing countries in transition and witnessing rapid urbanization. A health club is sustainable and culturally appropriate when facilitated by community or local people who have an insight and a deeper understanding of the culture and environment of the people they serve. Although a health club can be used as a platform to facilitate lifestyle changes, an enabling environment is necessary for the maintenance of healthy living. Therefore, in order to adequately address the problem of hypertension and other NCDs in disadvantaged communities, there is a need to focus on social determinants of health. 

## Figures and Tables

**Box 1:**
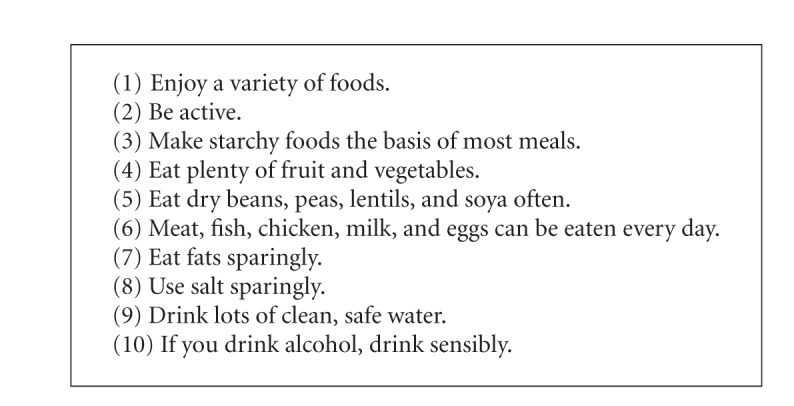
Food-based dietary guidelines.

**Box 2:**
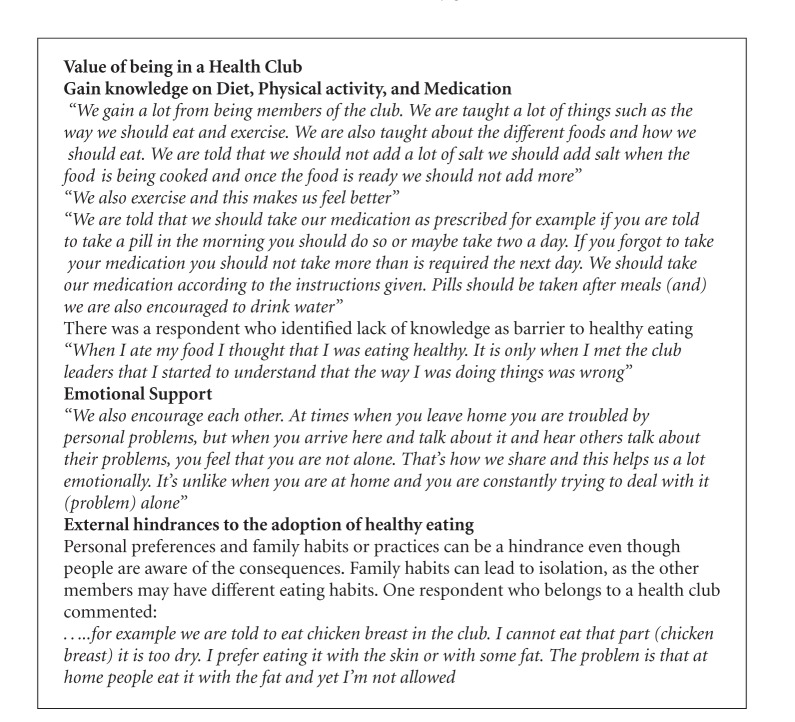
Testimonies of health club participants.

**Table 1:** General characteristics of club members.

Variables	Inception of the health club (*n* = 22)	2 years after inception (*n* = 30)
Gender		
Males	6 (27.3%)	3 (10%)
Females	16 (72.7%)	27 (90%)
Mean age (years)	50	61
Age range by gender (years)		
Males	43–65	50–73
Females	30–61	38–87

**Table 2:** Anthropometric measurements and mean values of the participants.

Variables	Inception of the health club (*n* = 22)	2 years after inception (*n* = 30)
Mean weight	82.7114	80.33
Range of the weight (kg)	52.95–118	55.56–133.9
Mean BMI	32.1899	31.62
Mean systolic blood pressure (mm Hg)	140	151
Mean diastolic blood pressure (mm Hg)	86	86
